# The Role of Veterans Affairs in Emergency Management: A Systematic Literature Review

**DOI:** 10.1371/198d344bc40a75f927c9bc5024279815

**Published:** 2012-12-12

**Authors:** Maria Claver, Darya Friedman, Aram Dobalian, Karen Ricci, Melanie Horn Mallers

**Affiliations:** Veterans Health Administration Emergency Management Evaluation Center and VAGLAHS HSR&D Center of Excellence for the Study of Healthcare Provider Behavior, California State University, Sepulveda, California, USA; Veterans Health Administration Emergency Management Evaluation Center and VAGLAHS HSR&D Center of Excellence for the Study of Healthcare Provider Behavior, California State University, Sepulveda, California, USA; Veterans Health Administration Emergency Management Evaluation Center and VAGLAHS HSR&D Center of Excellence for the Study of Healthcare Provider Behavior, California State University, Sepulveda, California, USA; California State University, Fullerton, California, USA

## Abstract

The Department of Veterans Affairs (VA) is a key player in emergency management for both veterans and civilians. Unfortunately, limited evidence-based research findings exist regarding the role and experience of VA during disasters. The present study is a systematic literature review of 41 published, peer-reviewed articles regarding VA and emergency management. Trained researchers utilized a data abstraction tool and conducted a qualitative content analysis. A description of article characteristics include methodology, phase of emergency management addressed in the research, and study design. Five topic categories emerged from the review including effects of disaster on mental health status and services use, effects of disaster on general health services use, patient tracking, evacuation, and disaster planning/preparation. Findings were used to generate suggestions for future research. Keywords: Veterans Affairs, veterans, disaster, emergency

## BACKGROUND

In addition to its primary mission to provide health services for veterans, the Department of Veterans Affairs (VA) is responsible for assisting federal agencies and the general public during emergencies as directed by the 1982 VA/Department of Defense Health Resources Sharing and Emergency Operation Act (Pub. L. 97-174). Given its nationwide presence, VA must respond to the range of emergencies and disasters through participation in the mitigation, preparedness, response, and recovery stages of emergency management. It does so, in part, through collaboration with other governmental, non-profit, and for profit agencies.

Collaboration requires effective communication through information dissemination. Existing research outside of VA does not adequately address the needs of those in disasters or VA’s activities in assisting its partners. Information about VA and emergency management is most often found in VA internal “after action reports” and is not widely disseminated. There is an urgent need for evidence-based research related to disaster and emergency response processes that VA is likely to engage in. This review thus aimed to explore and summarize the current scientific literature about VA and emergency management and make suggestions for future research.

## METHOD



The first step included searching for relevant emergency management peer-reviewed articles published between 1982 and 2012. RAND Corporation librarians used disaster-related search terms using relevant academic databases (see Table 1) to provide the research team with a list of articles (n=877) that met initial search criteria (i.e. contained search terms mentioned above in the title, keywords, or abstract). Of the 877 articles, 798 were rejected at title review as clearly irrelevant to the project or were excluded as they were not published in peer-reviewed journals. This left 79 peer-reviewed articles that were then reviewed using the following inclusion criteria: 1) the article discussed emergency management, and 2) the article discussed services directly provided by VA, services for which VA contracted from outside entities, or veterans served by VA. Thirty-nine articles met the criteria. The reference lists for these articles were then reviewed for possible inclusion; all but 5 articles were rejected at title review. Of the 5, only 2 met the full inclusion criteria. Thus, 41 total articles were included in the final review for meeting criteria (Figure 1).

**Literature Search Flow:**
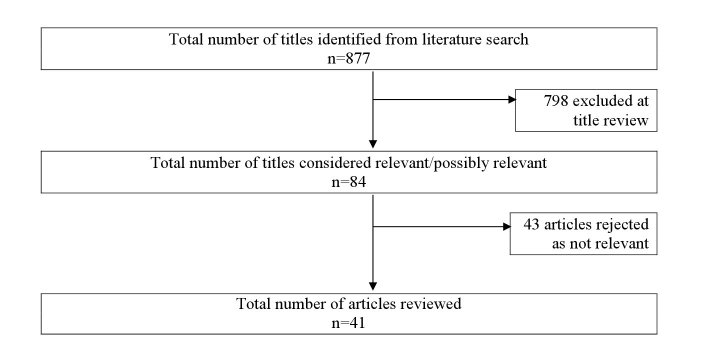

All of the articles were then fully reviewed using a data abstraction instrument designed for this study and reviewed by the VA/UCLA/RAND Southern California Evidence-Based Practice Center (Figure 2). The data abstraction instrument categories were compiled from literature reviews, pilot testing of the instrument, and input from experts in emergency management research and practice. The categories on the data abstraction instrument reviewed in the current study included: 1. Study design (e.g., experimental including randomized groups and a control group, quasi-experimental including comparing different groups under different conditions such as time periods or regions, or pre-experimental including examination of one group over time, retrospective reporting, data tracking, program evaluation or descriptive case studies); 2. Methodology (i.e., qualitative, quantitative, mixed); and 3. Role of VA in emergency management (i.e. mitigation/prevention, preparedness, response, recovery). Two members of the research team independently reviewed the articles and compared data coded on the data abstraction instrument. In cases where coding diverged, the team obtained reliability by reaching consensus about the code or via input from a third team member. Finally, the articles were reviewed using content analysis techniques 1 . The primary topic of each article was characterized with a code word or phrase. Articles pertaining to similar topics were then grouped together into categories described below.

**Data Abstraction Tool:**
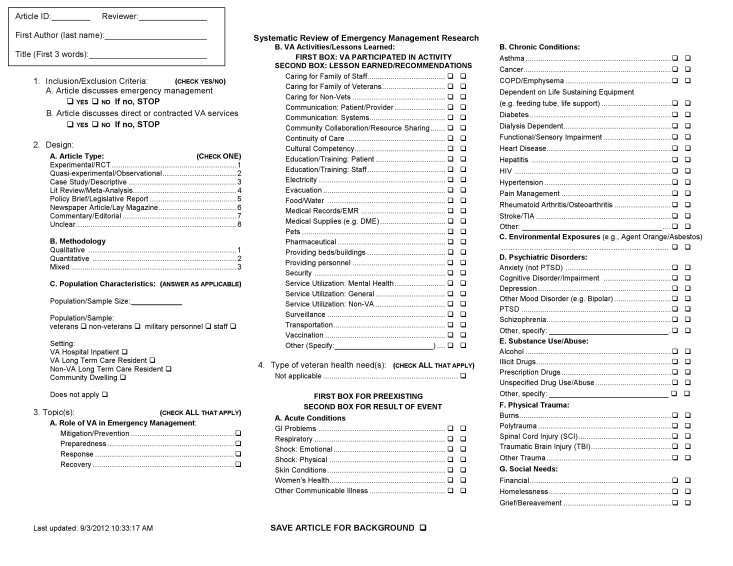

## RESULTS

The study design of the final group of articles was experimental (n=0), quasi-experimental (n=12) or pre-experimental (n=29). Methodologies included qualitative (n=21), quantitative (n=19), and mixed (n=1). The role of VA in the emergency management phases included mitigation/prevention (n=5), preparedness (n=2), response (n=8), or recovery (n=14). Several articles (n= 12) addressed more than one emergency management role, including two articles that addressed all four roles.

Five topic categories emerged from the review: Effects of disaster on mental health status and services use, effects of disaster on general health services use, patient tracking, evacuation, and disaster planning/preparation.

**Effects of Disaster on Mental Health**

Thirteen articles relate to the effects of disaster on mental health, including service use and status. Among studies that explored mental healthcare use for PTSD symptoms 2 3 one study 2 found that the use of non-VA healthcare was likely to increase following 9/11, while another 3 found no significant increase in VA or non-VA mental health services by those with previously diagnosed PTSD. Others 4 5 reported a decline in mental health and substance use services following Hurricane Katrina, especially for those with PTSD. One possible explanation 3 given for this decline in service use, despite its availability, was that numbing and avoidance are symptoms of PTSD.

Regarding mental health functioning, veterans treated in PTSD clinics experienced fewer symptoms at admission after 9/11 than veterans admitted before 9/11, contrary to expectations 4. Moreover, these veterans showed further improvement as time went on after 9/11. Longitudinal research conducted among veterans pre- and post-9/11 shows that PTSD symptoms were exacerbated immediately following 9/11, including avoidance/numbing and hyperarousal, but that these were not sustained over time 6 . In addition, research on the subjective experiences of veterans indicate that their thoughts and feelings about 9/11 affected their overall functioning 7. Finally, regarding self-reports, people are generally consistent about reporting traumatic events, particularly events related to PTSD 8.

In addition to examining PTSD, several studies considered general mental health issues. Following 9/11, there were increases in unscheduled clinic visits by veterans for symptoms of anxiety and depression 9, which are linked with PTSD 10. Further, veterans who experienced potentially traumatic events (PTEs) prior to Hurricane Katrina had increased likelihood of experiencing violent PTEs during Katrina 11. Similarly, individuals with pre-Katrina mental illness showed higher levels of post-Katrina cognitive bias, including negative interpretations and emotional responses to the disaster 12. Finally, an examination of emotional distress among veterans following the 1994 Northridge Earthquake 13found that, similar to the above findings, pre-disaster emotional status was a strong predictor of individual perception of earthquake impact, as well as post-disaster distress. Findings make salient the impact of disaster on vulnerable populations 13 14, especially veterans of low socioeconomic status and minority groups. Overall, these studies point to the importance of outreach and service provision to disaster survivors who struggle with or are at risk for emotional illness following disasters.

**Effects of Disaster on General Health Services Use**

Eight studies address general health services use affected by disaster. From the provider’s perspective, it is challenging to provide continuity of care because of several barriers including limited or compromised communication infrastructure 15, lack of records 15 16 and uncertainty regarding best practices 15. Characteristics such as interdisciplinary collaboration and support of medication counseling from VA and non-VA local pharmacists have been shown to mitigate the challenges 15.

Despite challenges and uncertainty, VA facilities, mobile clinics, and a VA Disaster Application Center (DAC) successfully served veterans and non-veterans alike in the first days following a disaster. Mobile clinics were effective because veterans favored receiving medical care in their communities, but the availability of care through mobile clinics presented the challenge of treating non-routine populations, such as infants and children. Volunteer practitioners should be thus versed in treating pediatric patients and knowledgeable about types of post-disaster conditions they would likely treat through the mobile clinic and DAC 16 17. Despite reports of effective response immediately following the disaster, there was a call for increased provision of follow-up healthcare services after the first 72 hours following disaster 15 16 18. One example is the Acute Traumatic Stress Management Protocol, which emphasized primary prevention for survivors of a disaster (e.g., immunizations to protect victims from communicable diseases) 15. Irrespective of the care provided, it is argued that healthcare providers should engage in evidence-based practice to avoid perpetuating misperceptions of unnecessary healthcare needs by the public due to uncertainty caused by a disaster 19. In addition, chronic pain and diabetes management services 20 21 and services for veterans with spinal cord injuries and disorders 22 were affected by considerable barriers after a disaster highlights the need for better coordination between clinics and specialists.

**Patient Tracking**

Three articles emphasize the importance of emergency medical records for optimizing service provision. One case study 23 describes the paper-based medical record system employed after the Northridge Earthquake used by all of the medical stations, disaster action centers and mobile clinics deployed after the earthquake. The authors attribute their ability to provide care to more than 11,000 disaster victims to an organized system that used simple forms, was consistent across providers and locations, had a central data collection point, made records available and accessible to providers at a known site every 12 hours and required the transfer of medical records with the patient. Prior to the Northridge earthquake, published information about data collection during a disaster was particularly sparse. Ten years later the VA Computerized Patient Record System (CPRS) was implemented, technology that made it possible for every VA provider across the nation to access any veteran’s health care records, was released and is credited with making the VA response to Hurricane Katrina a successful one. CPRS supported continuity of care because the electronic medical records included not only prescriptions and lab results, but also case notes and other documents critical to care provision 24. Despite the widespread adoption of CPRS throughout VA, electricity and computers are not always available at the site of a disaster. A pharmacist describes his experience of serving victims of the Northridge earthquake without computer (and CPRS) access until two weeks following the event 25. He describes a process of keeping a logbook and handwriting medication labels until CPRS became available. These articles serve as a reminder that ongoing tracking of patients before and after a disaster plays a key role in successful response.

**Evacuation**

The five case studies about evacuation following a major disaster share several characteristics and lessons learned while each report includes experiences unique to the particular evacuation. There is a clear need for prompt decisions made by the administration, coupled with an understanding of the chain of command by all involved in the disaster response 26. In some cases, administrators may not be available, so staff should be empowered to make decisions, if necessary. Moreover, the normal chain of command needs to be modified in the case of a disaster 27. There was a call for a redefinition of “essential personnel” during a disaster, placing emphasis on the importance of facilities management for tasks such as laundering and those with experience arranging transfer of patients, such as social workers, case managers and medical administrators 28. The availability of manpower was discussed as critical to the success of an evacuation 25 29.

Working with the community was another topic of importance within evacuation. Pre-existing agreements with community resources and hospital facilities that could host evacuated veterans was cited as critical to the evacuation 26 27, as was on-the-spot collaboration with local entities, such as the fire department and other medical centers 29. It was noted in one case 26, though, that despite pre-arrangements with local hospitals, the plan was negated by local city government. The unavailability of means of communication may have hampered use of community resources. A loss of or hindered communication was mentioned in all of the articles as problematic during evacuation. In particular a loss of emergency medical record access through VA’s CPRS, which compromised access to real time data, was noted.

Continuity of care, as shown in two studies, was not compromised because providers made an effort to follow patients that had been transferred to another hospital due to a loss of utilities and potential explosion 26 and internal flooding 29. Challenges to continuity of care in these and other disasters included pain management 27 and providing care despite adverse weather 26 27 and loss of water and power 28.

All of the studies detailed lessons learned and modifications in disaster planning that have been made since the disaster. These include increased training, stocking indoor supplies, and the development of mitigation teams that supervise improvements in disaster preparedness 27. The development of plans should take into account the need for prolonged recovery above and beyond the acute disaster event 28, though plans should be "flexible enough to allow for creative, on-the-spot problem solving" [297, p.156].

A disaster response framework 30 makes salient the need to provide comprehensive care during evacuation, especially for vulnerable nursing home residents. The proposed framework was designed to address the comprehensive needs of patients during evacuation, including the role of the community, as well as highlight the significance of communication and collaboration with federal, state, and local emergency entities. Similar to the above articles, it addresses the role of the community and coordination of services necessary for successful short-term and long-term evacuation-related outcomes that moved above and beyond temporary evacuation to consider the need for permanent evacuations, especially for vulnerable nursing home residents. Overall, all of the articles point to the need for continued evidence-based studies on evacuation preparedness and response.

**Disaster Planning/Preparedness**

Twelve of the included articles are about disaster planning and preparedness. These articles covered a wide variety of emergency management topics, though a common theme was the importance of collaboration and education 32 for improving overall response to future disasters. One article articulated that part of effective planning and preparation is the inclusion of all healthcare providers in disaster response, such as pharmacists 34; another articulated the critical need for including clear content related to disaster communication, providing guidance from national organizations, and following predetermined communication plans 35. Other articles provided specific examples of effective collaboration using Biosense 31 33 36, a paradigm for public health surveillance that collects and analyzes secondary data for bio-surveillance purposes, and can be used to effectively connect the VA with other agencies. Lastly, innovative approaches to track data predictive of disease outbreaks were discussed 37 38, including a VA national electronic biosurveillance system to track cases of influenza 38 and usage of VA employee sick-days 37.

Additionally, three articles highlight the importance of VA to ensure its internal effectiveness related to disaster to be able to continue providing emergency assistance to the wider community 39 40 41. For example, it is important that during mass-casualty decontamination programs, VA continues primarily to protect itself so that it can maintain continuity of health care to veterans and to respond to civilian needs 39. Another means by which VA can effectively protect veterans is to design educational campaigns about disaster planning that meet the specific needs of veterans and their families 40. Lastly, recommendations for an increase in risk evaluation and training regarding the security of radioactive material at VA facilities were presented 41. Overall, these articles indicate the importance of maintaining a strong VA system, which can then foster strong ties with other agencies and communities 42, thus allowing for opportunities to share successful planning practices for decreasing disaster-related risk.

## CONCLUSION

This literature review identified and synthesized 41 peer-reviewed articles about VA-related emergency management. The majority of articles were pre-experimental. While this affords us with detailed case studies about specific disasters, more experimental research would be beneficial. For example, evidence-based prevention programs for PTSD and other mental health issues following disaster could greatly add to the stress and coping literature on veterans and serve as the foundation for the development of effective intervention. Similarly, few studies used mixed method design. More studies on emergency management that integrates qualitative and quantitative data will afford the field with a more coherent and complete picture of its strengths and gaps. Additionally, among the phases of emergency management, there is need for more work particularly on preparedness. While twelve articles mentioned some aspect of preparedness, only two articles specifically addressed plans to handle an emergency, calling for an increased need of effective communication practices. We suggest more studies on the processes necessary for effective community collaboration and development of plans and protocols to prepare for possible emergencies.

Overall, five themes emerged from this review. The first is the effects of disaster on mental health status and services use. This group of articles point to a need for ongoing longitudinal studies on mental health impact due to disaster, as well as increased outreach efforts to victims. Also, only a few studies examined individual differences in mental health impact (e.g., ethnicity). This gap could be remedied with increased research efforts related to psychosocial risk factors and individual’s vulnerability to disaster. We suggest including ethnicity, SES, gender, and age as key variables in future research.

The second theme, effects of disaster on general health services use, highlighted the need for continuity of care. Several barriers were identified including poor communication infrastructure, although effective collaboration, such as with pharmacists, was shown to mitigate such challenges. Hospital administrators and public health officials would greatly benefit from an inclusive approach to communication practices. Related articles documented patients’ preference for receiving treatment in their community, such as through mobile clinics. This makes salient the need for support and camaraderie in disaster recovery. The need for ongoing discussion about planning for both short-term and long-term health care services was highlighted, as was greater understanding of the specific needs of the community being served.

The third theme, patient tracking, sheds light on the need for systematic protocols to organize data on patients receiving care following a disaster. Examples of successful processes were shared. These articles serve as a necessary reminder of the critical need of tracking for patient survival following disaster.

The fourth theme, evacuation, indicates an overall need for more evidence-based research, including decision-making and the roles of all personnel during disaster, including plans for ongoing collaboration and communication with city officials and local hospitals, especially as it relates to “back-up” evacuation plans. Efforts to develop plans for long-term evacuation were also deemed as critical. Careful consideration also needs to be given to identifying best practices for evacuation practices for specific, unique populations being served. Community needs assessments prior to and following disaster are thus recommended.

Articles related to disaster planning/preparation shed light on the importance of collaboration. This is particularly necessary as it relates to interrelationships between the VHA and community partners during local emergencies and federal disasters. This is also critical as it pertains to ongoing dissemination of research findings related to disaster experiences. With increased collaboration and communication, overall disaster planning and response mechanisms can be strengthened. Interestingly,this call for action reflects the mission of the Evidence Aid project 43 44 which grew out of The Cochrane Collaboration, an international network linking together healthcare providers, policy makers, patients and their advocates worldwide in an effort to improve health-care decisions. This is achieved in part through The Cochrane Library, which houses on-line databases of literature and research, including randomized controlled trial studies and systematic reviews, on health care practices. Through dissemination of high-quality research on disaster health problems, we can support interested parties to timely and cutting-edge information on best practices that will likely improve the quality of life of those affected by disaster.

Finally, while additional research is needed within each theme identified, it is also important to note that these themes are not mutually exclusive; rather they influence each other. For example, increased tracking of services following disaster directly informs decision-making regarding service provision. Similarly, continuity of care is not merely a challenge within general health services usage, but should also be a topic of discussion within all phases of emergency management for both general and mental health services usage. An integrated, comprehensive approach to research will provide a clearer picture about the role of VA in caring for veterans during times of disaster and also about VA as a collaborator that provides support to the community before, during and after a disaster.

## Competing Interests

The authors declare the no competing interests exist

## Correspondence

Corresponding Author: Maria Claver (mclaver@csulb.edu)

## Appendix 1

### Articles included in the systematic literature review

Download PDF (50KB)

## Appendix 2

### PRISMA Checklist

Download PDF (85KB)
